# Additively manufactured, long, serpentine submillimeter channels by combining binder jet printing and liquid-phase sintering

**DOI:** 10.1038/s41598-024-65058-5

**Published:** 2024-07-22

**Authors:** Truong Do, Hawke Suen, Aryan Mehboudi, Tyler Bauder, Christopher Rudolf, Patrick Kwon, Junghoon Yeom

**Affiliations:** 1 College of Engineering and Computer Science, VinUniversity, Hanoi, Vietnam; 2 Department of Mechanical Engineering, Michigan State University, East Lansing, MI 48824 USA; 3 Department of Mechanical Engineering, University of Texas, Austin, TX USA; 4 Code 6354, Materials Science and Technology Division, Naval Research Laboratory, Washington, DC 20375 USA

**Keywords:** Joining, Binder jet printing, Microchannel, Additive manufacturing, Depowdering, Sintering, Engineering, Materials science

## Abstract

Metallic microfluidic devices made from powder-bed additive manufacturing systems have received increasing attention, but their feasible channel geometry and complexity are often limited by lack of an effective approach to removing trapped powder particles within the channels or conduits of the sintered parts. Here, we present an innovative approach to fabricating long serpentine, high-aspect-ratio submillimeter channels made of stainless steel 316L (SS) by binder jet printing (BJP) and liquid-phase sintering. We leverage the unique nature of the BJP process, that is printing and consolidation steps are decoupled, enabling us to join two or more parts during the sintering step. Instead of constructing the channel device as a single part, we print multiple parts for easy powder removal and later join them to form enclosed channels. The key innovation lies in adding sintering additives like boron nitrides (BN) to the SS stock powder—at the SS/BN interfaces, liquid phase is locally generated at temperature much lower than the SS melting temperature, facilitating the bonding of the multiple parts as well as the consolidation of parts for near-full density. We systematically vary the sintering temperature to show how it affects the joining quality and the channel shape distortion. The joining quality such as the fracture strengths of the joined samples is measured by a pull test while the shape distortion is characterized by various imaging techniques. The feasibility of the proposed approach is demonstrated by fabricating a 400-mm-long, fully enclosed serpentine channel with a rectangular cross-section of 0.5 mm in width and 1.8 mm in height. The pressure drop across this 3D-printed SS serpentine channels is measured for air flow and compared to a standard gas flow model, showing that the device is free of leakage or clogs.

## Introduction

Serpentine micro- or submillimeter channels are commonly found in the microfluidic circuits^1,2^, mixers^3^, separation columns^4,5^, and heat exchangers^6,7^ mainly because a serpentine pattern enables the dense packing of channels on a planar substrate. The densely packed microchannels are desirable as they provide more surface areas for reaction, retention, and heat/mass transfer on a given planform area^8^. But these benefits come at a price of higher pressure drop and more pumping power^9^. One approach to mitigating the issue of high pressure drop in a long serpentine channel is to make the channel deep, effectively increasing the hydraulic diameter and yet maintaining its dense packing. Silicon serpentine microchannels of an aspect ratio greater than 5 were fabricated by anodically bonding a deep reactive ion etched Si die with a glass plate^10,11^. Such high-aspect-ratio channels are, however, limited to only Si-based microfluidic systems and unavailable for other common microfluidic structural materials such as glass and polydimethylsiloxane (PDMS)^12^ partly due to lack of proper fabrication methods.

Conventional machining techniques such as micro-milling^13^, laser milling^14^, micro-electrical discharge machining^15^ have been employed to fabricate microchannel-like grooves in various metallic substrates^16^. Among these techniques, micro-milling has been preferred for creating serpentine micro- and submillimeter channels in metals^17^. However, as the groove depth or the channel aspect ratio increases, common milling issues such as top burr formation, tool wear, and surface roughness become exacerbated^18,19^. More importantly, once channel-shaped cuts are made, the machined surface needs be bonded or welded to another piece to form a fully-enclosed channel device, necessitating an additional step that is not easily accomplished at small scales. Recently, additive manufacturing (AM) offers unique opportunities for creating metal parts with more geometric freedom, and fully-enclosed metallic microfluidic devices made from various AM techniques have received increasing attention^20^. Some notable examples of such devices include conformal cooling channels for mold^21–23^, heat exchangers^24,25^, microreactors^26^, and micro gas chromatography^27–29^. Most of these AM-based metallic submillimeter channels were fabricated using powder bed fusion (PBF) systems such as selective laser sintering (SLS), selective laser melting (SLM), electron beam melting (EBM), and binder jet printing (BJP).

One of the main challenges in generating a part with fully-enclosed micro- or submillimeter channels via PBF is to remove trapped powder from the printed channels^30^. The channel parts printed from SLS, SLM, or EBM are consolidated and strong, allowing sand blasting, compressed air, or vacuum suction to be employed to remove the un-sintered loose powder particles within the conduits. However, when the channel parts include multiple bends or have a small characteristic length (i.e., diameter) and/or a long channel length, the efficiency of removing trapped powder is significantly undermined^31^. Therefore, 3D-printed metallic serpentine channels are typically limited to open structures^32^. Removal of the trapped powder particles in BJP is even more problematic because the green part state is most fragile^33^. Vlasea et al.^34^ invented a hybrid process based on the BJP system that has an ability to fill the channel with a sacrificial photopolymer which was later burned to leave the internal channel area open. But with this method, it is difficult to control the channel shape, which is often limited to a circular channel.

Here we present a novel approach to fabricating long serpentine, high-aspect-ratio submillimeter channels made of stainless steel (SS) by means of BJP and liquid-phase sintering (LPS). Instead of building a microfluidic component as a single part, we propose to print separate parts and later join them while being fully sintered. Parts that form channels can be designed and printed in two or more entities, where certain surfaces of one entity can be designed to be opened for easy powder removal. Unlike the other powder bed systems, BJP decouples printing and sintering steps, and during the final sintering step, in addition to obtaining near full-density, multiple parts can be joined—a process similar to the metallurgical joining or bonding via LPS^35,36^. Therefore, we argue that no additional steps are needed to build the proposed submillimeter channel device as opposed to SLS, SLM, or EBM where printing and sintering take place at the same time and joining parts entails an extra step. The proposed approach is built on our earlier works on the modified BJP process combined with LPS to attain near full-density (> 99%) SS parts for SS420^37^ and SS316L^29,38^. The key enabler for our modified BJP as well as the proposed joining process is to add a small amount of sintering additives like boron compounds to SS stock powder. Boron compounds locally form a liquid phase only at their interfaces with the SS powder at temperatures substantially lower than the melting point. This facilitates bonding at the interfaces of the parts as well as their consolidation for near-full density. To demonstrate the feasibility, we will fabricate a two-story, 400-mm-long fully-enclosed serpentine channel with the rectangular cross-section of the width of 0.5 mm and height of 1.8 mm. We will also show how small changes in key process parameters such as sintering temperature and duration can influence the joining quality like the interfacial strength and final channel geometry. The joining quality will be characterized by mechanical measurements as well as by electron microscopy and micro-CT. Finally, we will demonstrate the possibility of 3D-printed SS serpentine channels as a microfluidic device by measuring the pressure drop across the channel and comparing it to the standard gas flow model.

## Materials and methods

### Materials

Stainless steel (SS) 316L powder of average size 14 μm purchased from Oerlikon Metco, Inc. (Troy, MI, USA) was used for stock materials. The default powder size recommended by the BJP manufacturer, ExOne, is 30 μm, but in this paper, we used a smaller particle size to print parts with finer feature sizes. The details of the powder characteristics such as size distribution and elemental analysis were reported in our earlier works^37,38^. To enhance the diffusion process required for joining and reduce the liquidus temperature for full sintering, 0.5 wt% of boron nitride (BN) powder with the average size of 1 μm (Sigma-Aldrich, St. Louis, MO, USA) was added as a sintering additive to SS316L powder^39^.

### Channel design

**Figure 1:**
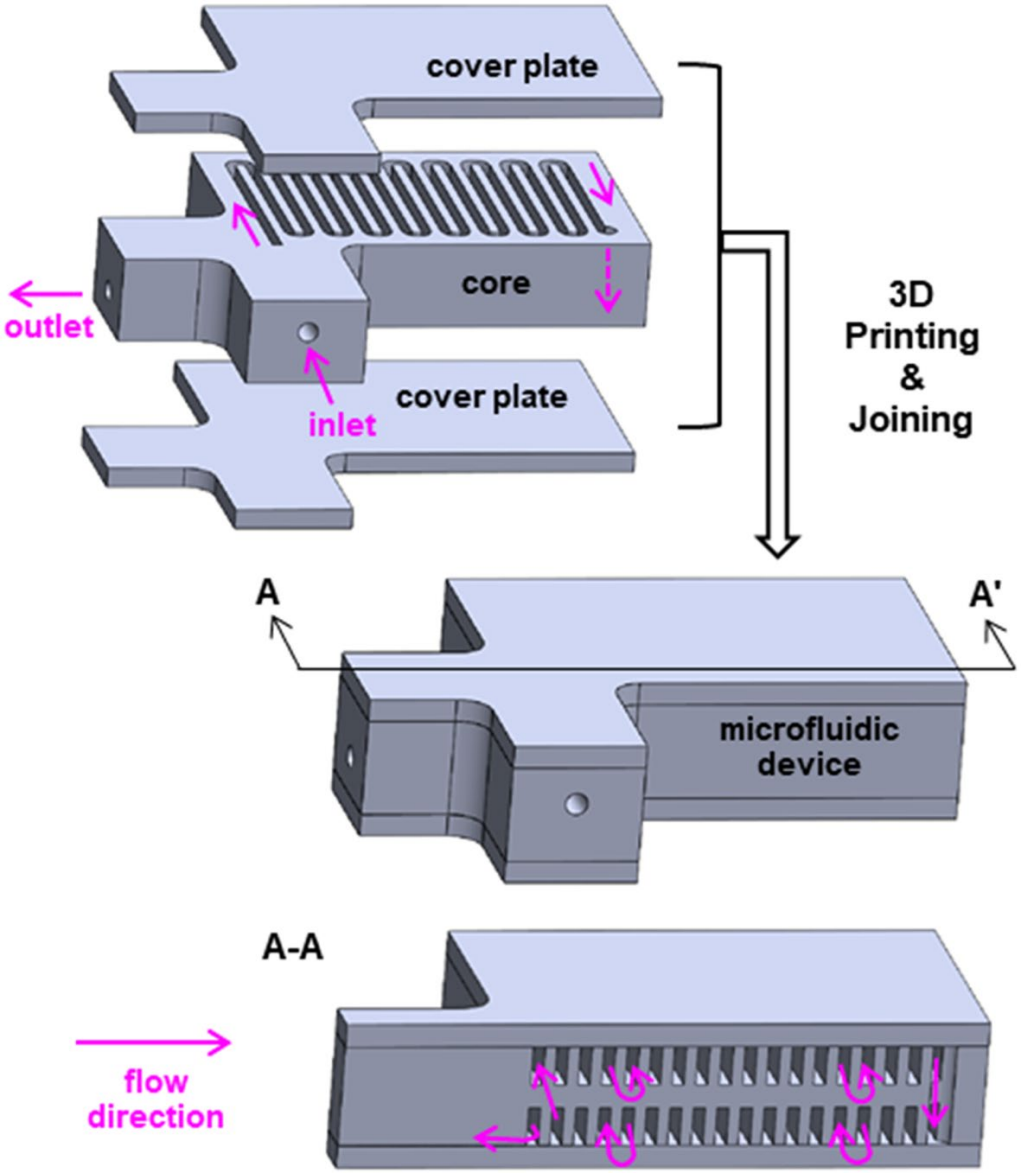
Schematic diagrams of the proposed serpentine submillimeter channel device, consisting of two flat cover plates and a core part with serpentine grooves on both sides. Two serpentine channels are stacked to double the effective channel length for a given planform area (pink arrows indicating a flow direction).

To test the feasibility of fabricating a long, high-aspect-ratio serpentine channel, we devised a two-story, 400-mm-long serpentine channel with the rectangular cross-section of the nominal width of 0.6 mm and nominal height of 2 mm (see the schematic diagrams in Fig. 1). The nominal wall thickness of the channel was 0.6 mm. In general, BJP parts shrink substantially after full sintering, and thus it is important to consider such shrinkage in the design phase. The channel width was designed about 20% larger than the target dimension of 0.5 mm to account for shrinkage that occurs during the sintering step of BJP parts. We chose to print three separate parts—a core part containing open serpentine channels on both sides and two cover plates for sealing and forming the enclosed channels as shown in Fig. 1. The top and bottom channel layers are 200 mm long each and connected through a via vertical channel. The vertically-oriented rectangular shape is selected as the channel cross-section to increase the channel packing density while reducing pressure drop. Powders occupying the serpentine channels can now be easily removed after printing but before joining/sintering. Additionally, to determine the minimum channel dimension, we designed another device with the varied channel widths of 0.2 mm, 0.3 mm, 0.4 mm, 0.5 mm, and 0.6 mm and the varied channel wall thicknesses of 0.3 mm, 0.45 mm, and 0.6 mm. The channel height was fixed at 2 mm (all dimensions are nominal). The same three-part approach, i.e., a channel core part sandwiched by two cover plates, was used to print a device assembly. Table S1 in Supplementary Information lists the nominal dimensions and sintering conditions of all channel devices considered in this study.

### Processing: printing and joining/sintering

**Figure 2:**
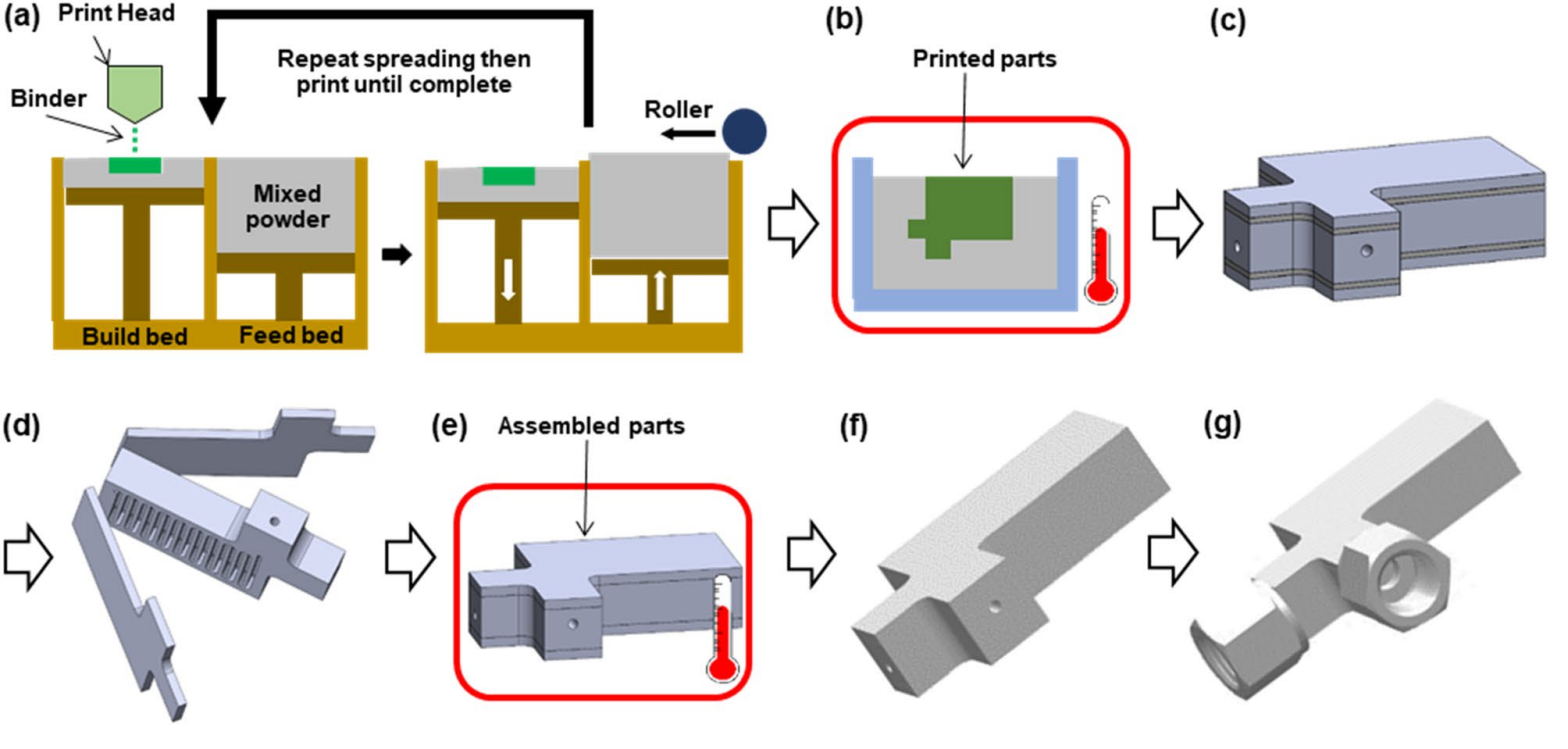
Step-by-step illustration of the proposed process steps for device fabrication using binder jet printing and diffusion bonding: (**a**) binder jet printing of three green parts—two cover plates and one core, (**b**) binder phase curing, (**c**, **d**) removal of loose powder, (**e**) binder phase burn-out, (**e**, **f**) full sintering and joining of the three parts to form the submillimeter channel device, (**g**) welding of fluidic interconnects.

The detailed BJP setup and printing processes can be found in our earlier work^37^. Briefly, two stock powder particles of SS316L and boron compound (in this case BN) were weighed in the mass ratio of 99.5–0.5% and well mixed in the high-speed mixer (DAC 150 manufactured by FlackTek, Inc., Landrum, SC, USA) in three cycles with an angular velocity of 2000 rpm and 90 s per cycle. The BJP process was conducted using the X1-Lab printer (ExOne, Huntington, PA, USA). The proprietary binder phase supplied by Exone Inc. was used with the default saturation level of 70%. Upon completion of printing (Fig. 2a), the build bed containing the printed parts and loose powder was transferred to a convection oven (DX302C, Yamato, Japan) to cure the polymeric binder phase at 195 °C for two hours (Fig. 2b). The printed parts became sufficiently strong for powder removal. For the two plates, the loose powder was easily removed by a soft brush. For the core part with the open channels, vacuum was used to draw out all loose powder (Fig. 2c,d). After removing loose powder, the printed parts were put into the air furnace (KSL-1100X, MTI Corp., USA) to burn out the binder phase for 2 h at 460 °C in air (Fig. 2e). The temperature was ramped up from room temperature to 460 °C at a rate of 5 °C/min, and after the soaking step, the temperature was brought back to room temperature at a rate of 5 °C/min.

After the binder burn-out step, the three parts were stacked in order on an alumina substrate (1/8″ thick, MSE Supplies, USA)—one cover plate at the bottom, the channel core placed in the middle, and the other cover plate on top. No external pressure was applied during the sintering. To reduce a potential interaction between the SS part and the alumina carrier substrate, a thin layer of yttria-stabilized zirconium oxide nanoparticles (20–40 nm, MSE Supplies, USA) was applied onto the alumina substrate. The stacked assembly was sintered at three different temperatures of 1130 °C, 1135 °C, and 1140 °C for 6 h (Fig. 2e,f). One set of the samples was sintered at 1135 °C for 12 h to see the effect of the sintering duration. The combination of argon gas and vacuum was introduced to purge the furnace chamber (Model G-3000, Materials Research Furnace, Allenstown, NH, USA) to keep the oxygen level at a minimum, and Ar was filled to the atmospheric pressure during the sintering step. The ramping rate used to increase to the target temperature and decrease to room temperature was 5 °C/min as stated above. The vertical channels in the core part were oriented towards the direction of gravity to minimize sagging or reflow during sintering.

### Image acquisition and analysis

The joined samples were then cut by a diamond saw to reveal the cross-section of the channels at a single location. The exposed channel cross-sections were polished before imaging. A camera (Nikon DS50, Japan) and scanning electron microscopy (SEM, Hitachi S-4800, Japan) were used to image the channel’s cross-section and joined interfaces. Measurement of the channel dimension was made using ImageJ software from the SEM cross-sectional images using at least 5 different channels and 5 different locations of each channel. The embedded serpentine channels were also imaged nondestructively using a lab-scale Xradia 520 Versa X-ray microscope (Carl Zeiss, Germany).

### Joining strength characterization

To qualitatively characterize the joining strength at the interface, we printed two identical parts of a rectangular prism (15 mm by 8 mm by 8 mm in size) and joined them in the lengthwise direction with the same sintering conditions as the channel samples, i.e., 1130 °C, 1135 °C, and 1140 °C for 6 h and 1135 °C for 12 h under the Ar gas environment. The joined blocks were sliced into thin bars with the thickness of 1 mm by Electric Discharge Machining (EDM, Brother HS-704, EDGE Machine, IL, USA). Three duplicate samples were fabricated for each sintering condition to ensure the repeatability of the measurements. To estimate the bonding strength, pull tests were performed on the bars in Instron Universal Testing System (3367 UTS, Instron, Northwood, MA, USA) with a pulling rate of 0.2 mm/min and a 30-kN load cell. Stress was calculated based on the applied load and the measured cross-sectional area of each joined sample. Table S2 in Supplementary Information lists the size and cross-sectional areas of the samples used for the strength characterization. Extension (or elongation) was measured from cross-head displacement because an extensometer could not be used due to the sample size constraints.

### Pressure drop measurements

As a non-destructive approach to investigate the overall quality of the fabricated channels with regard to existence/nonexistence of defects such as clogging, shortcut, etc., air flow through the fabricated device has been investigated theoretically and experimentally. Prior to the testing, two commercial hex nuts (1/16″ inch, McMaster Carr, USA) were welded on the channel inlet and outlet holes (Fig. 2g). The in-line pressure of air flow was adjusted using a pressure regulator (PneumaticPlus, PPR2-N02BG-4 Miniature Air Pressure Regulator). The volumetric flow rate of air was measured using a custom-built setup^40,41^. A small water plug was located within the outlet tube and pushed in and out depending on the applied pressure. The movement of the water plug was videotaped with a high-resolution camera, and the linear velocity of the water plug was measured to estimate the volumetric flow rate of air. Fluidic measurements were repeated four times to provide standard errors.

## Results and discussion

### Mechanical pull testing

**Figure 3:**
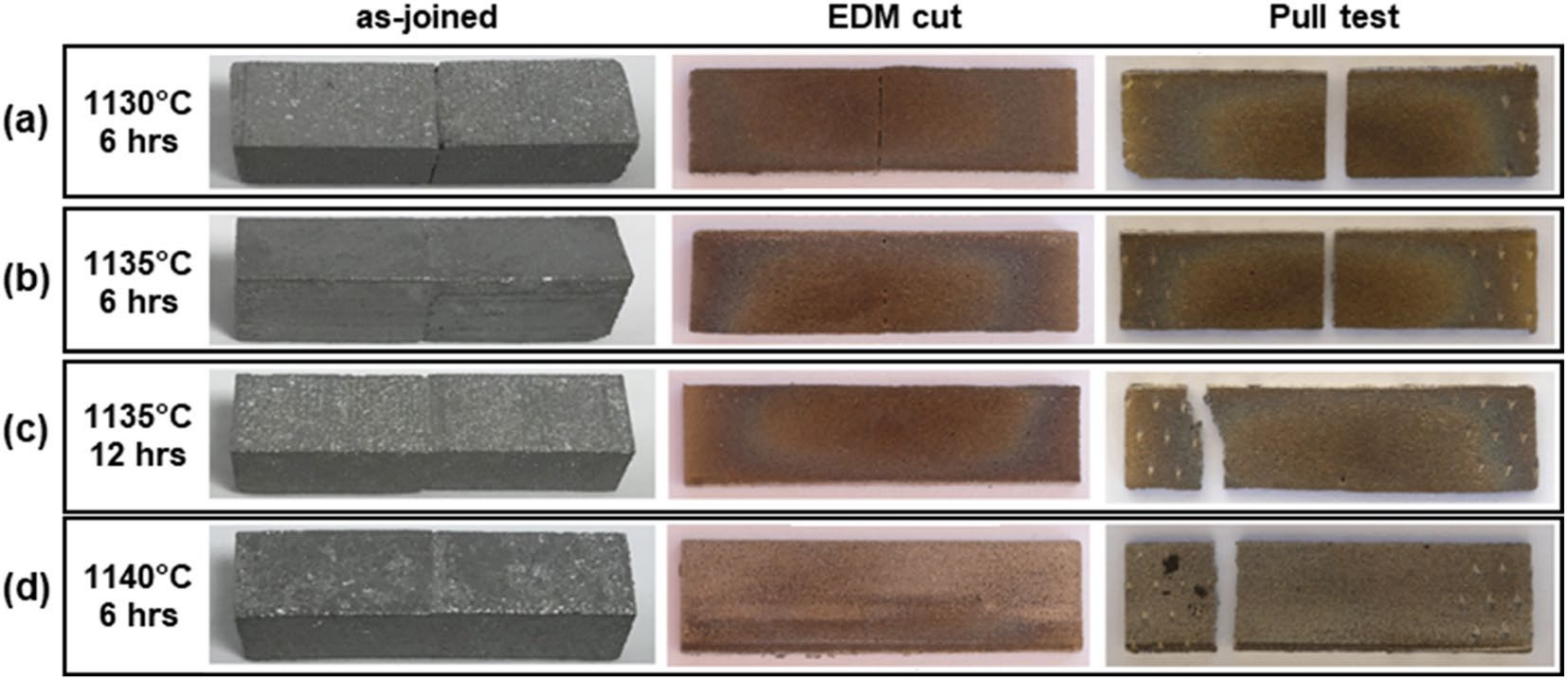
Photograph images of the four SS joined block samples at different post-processed stages: first column—as-joined of two rectangular bars (each bar with the dimension of 15 mm by 8 mm by 8 mm); second column—EDM cut of the joined sample; third column—after pull testing. Each joined sample has different sintering conditions (**a**) 1130 °C for 6 h, (**b**) 1135 °C for 6 h, (**c**) 1135 °C for 12 h, and (**d**) 1140 °C for 6 h.

Figure 3 shows the images of the four joined block samples at various process stages: as-joined, after EDM cut, and after the pull test. These four samples were sintered at different sintering conditions as described in the experimental section. The sample sintered at 1130 °C for 6 h shows the visible separation between two blocks at the joined interface (see Fig. 3a), suggesting that the sintering temperature may not be sufficiently high. As shown in Fig. 3b, the interfacial boundary of the two blocks sintered at the 1135 °C for 6 h is much harder to recognize. The 5 °C difference appears to make a significant improvement in bonding quality. The samples sintered at 1135 °C for 12 h and 1140 °C for 6 h show no visible bonding boundaries (see Fig. 3c,d).

**Figure 4:**
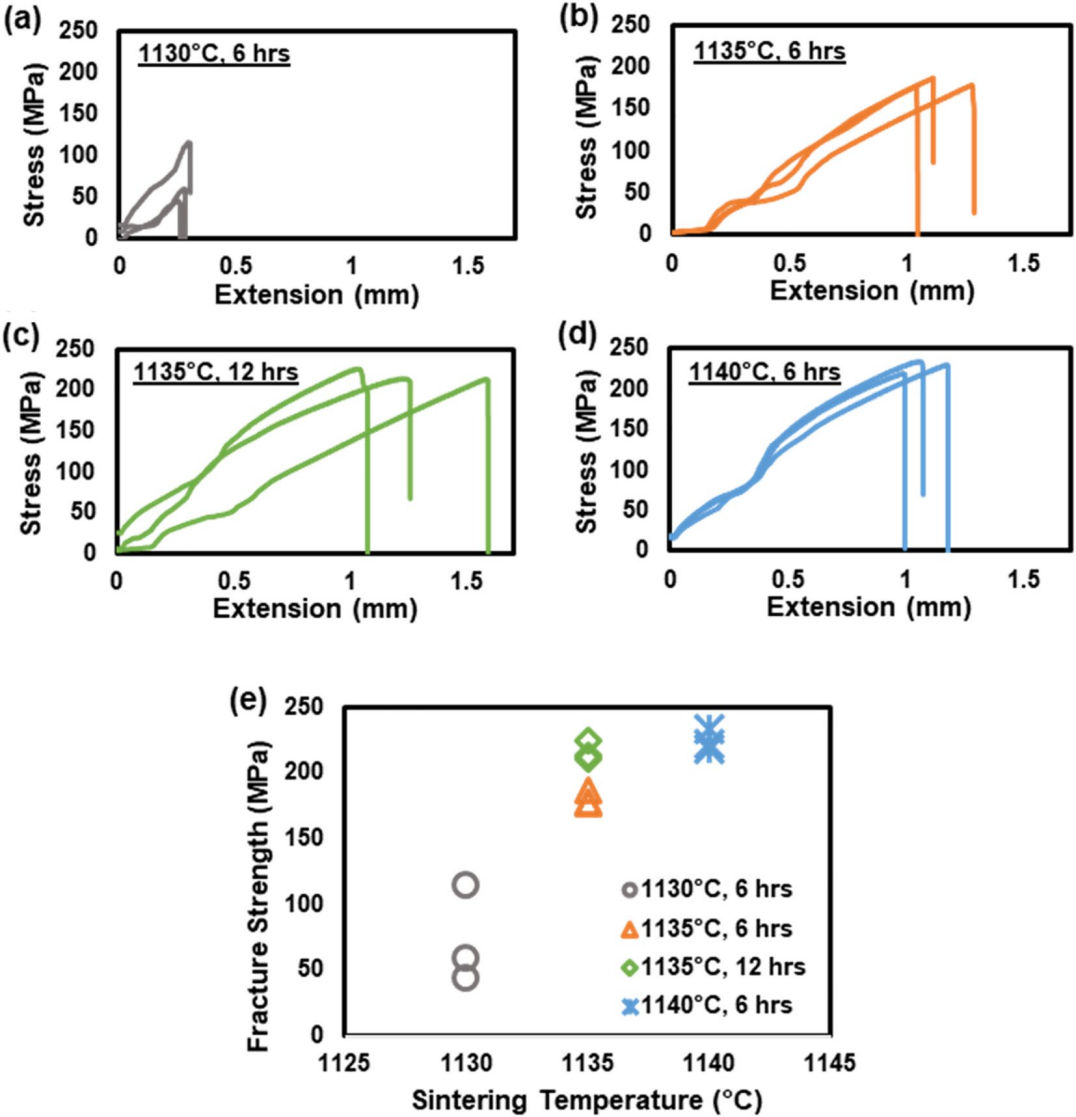
(**a**–**d**) Plots of the stress vs. extension of the SS joined parts (three duplicates for each condition) sintered at different temperatures (1130, 1135, and 1140 °C) and soaking durations (6 and 12 h); (**e**) Plot of the fracture strengths [maximum stresses at breaking points from (**a**–**d**)] as a function of the sintering temperature.

To verify if these observations are translated to the strength, we performed tensile testing on each joined sample prepared using the EDM cut. Three duplicate samples were made at each sintering condition for the statistical purpose, and the testing results are shown in Fig. 4a–d. Note that stress is plotted against elongation, not strain, as the sample is not a standard dog-bone-shape specimen with some slippages observed in the grip. Based on Fig. 4a–d, the fracture strength of each sample is plotted in Fig. 4e and estimated to be on average 72.7 MPa, 179.9 MPa, 216.4 MPa, and 224.2 MPa for the 1130 °C-6 h, 1135 °C-6 h, 1135 °C-12 h, and 1140 °C-6 h samples, respectively. The samples sintered at 1130 °C exhibit significantly lower strength compared with the others, which is consistent with the earlier observation made with the sample photographs. When the sintering temperature is 1135 °C or higher, the improvement in fracture strength is marginal. It is interesting to note that sintering the joined blocks for a longer duration made a noticeable difference in bonding strength, i.e., the 1135 °C-12 h sample exhibited about 40 MPa higher fracture strength than the sample sintered for 6 h at the same temperature.

With the fracture strength data in mind, we revisit Fig. 3 and focus on the locations of the failure points within the joined samples. While the samples sintered at 1130 °C and 1135 °C for 6 h failed at the bonding interface areas, the samples sintered at 1135 °C for 12 h and at 1140 °C for 6 h broke at the gripping locations with high stress concentration. This at least proves that the bonding strengths of the 1135 °C-12 h and 1140 °C-6 h samples are commensurate with those of the sintered parts themselves. This also implies that the sintering temperature higher than 1140 °C may not result in improved bond strength because the failure may occur within the parts, not at the joining interface. Previously, we measured the tensile strengths of the annealed SS316L specimen and the BJP-prepared/vacuum-sintered full-density SS316 part to be 515 MPa and 404 MPa, respectively^38^. Therefore, the average fracture strengths of the joined samples sintered at 1135 °C for 12 h and at 1140 °C for 6 h are about 42% of the fracture strength of the annealed SS316L sample and about 55% of the printed SS316L. The lower strength of the joined sample in this study in comparison to the BJP-printed sample (as a single part) of the earlier study^38^ can be attributed to the fact that the vacuum-sintered parts typically have a higher density (or lower porosity) than the samples sintered in Argon. Further research is needed to understand the failure mechanisms of the joined/sintered parts, particularly to determine whether porosity is the cause of the observed failure.

### Fabrication of serpentine submillimeter channel devices

**Figure 5:**
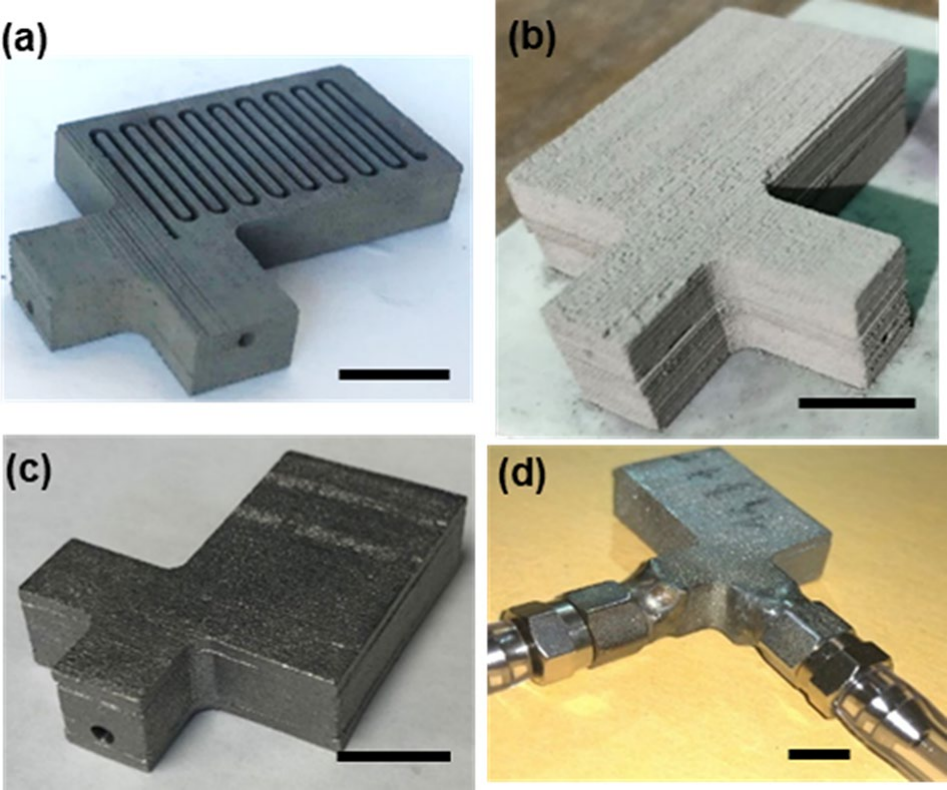
Photographs of the SS submillimeter channel device at each processing step: (**a**) after binder phase curing and loose powder removal, (**b**) after binder phase burnout, (**c**) after full sintering and joining, (**d**) after the fluidic interconnects welded. Scale bar in (**a**)–(**d**) = 10 mm.

Photographs of a SS316L submillimeter, serpentine channel device are shown in Fig. 5 for various processing stages. Loose powder particles occupying the channel volume were removed from the channel core part in its green state with the standard depowdering procedure such as soft brushing and vacuuming (see Fig. 5a). Comparatively, when the same channel device was printed in a single part, we could not remove the loose powder trapped in the channels. The core part was then sandwiched by two flat cover plates. When the assembly underwent the binder burnout process, the dark greenish assembly became light gray (see Fig. 5b) indicative of thin oxides formed on the powder surface. Upon full sintering under the Ar atmosphere, the assembly regained silvery shiny finish (see Fig. 5c). We previously reported that the surface finish quality of the fully-sintered BJP parts was improved by using the smaller powder size and adding sintering additives^37,38^. In this study, we employed the average size of 14 μm for SS 316L powder and 0.5 wt% of boron sintering additives, resulting in the average surface roughness of Ra ~ 8.9 µm. Figure 5d shows the fully-sintered/joined submillimeter channel device with the welded fitting and fluidic interconnect for pressure drop measurement.

**Figure 6:**
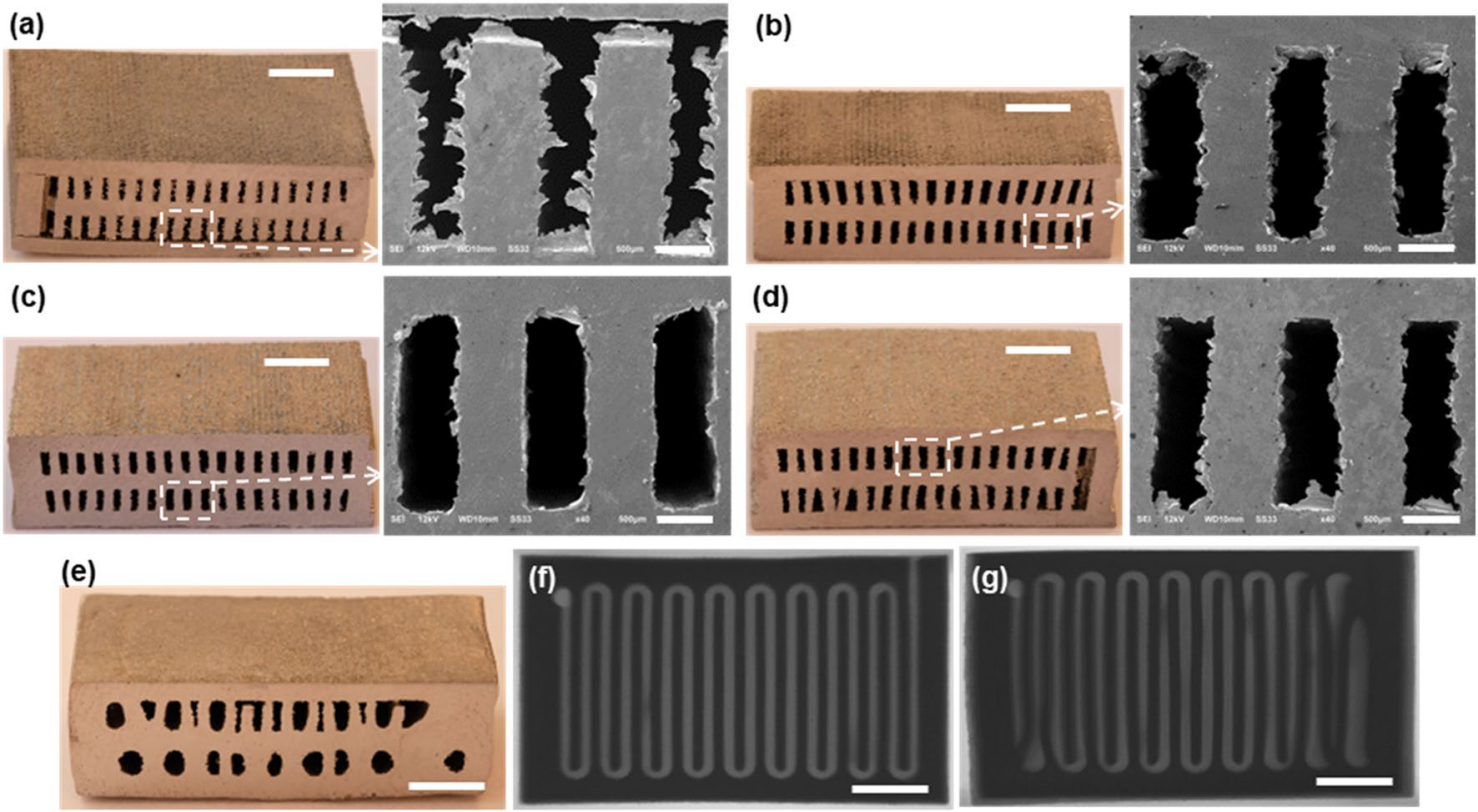
Photographs and SEM images of the SS submillimeter channel devices sintered and joined (**a**) at 1130 °C for 6 h, (**b**) at 1135 °C for 6 h, (**c**) at 1135 °C for 12 h, (**d**) at 1140 °C for 6 h, (**e**) at 1150 °C for 6 h; micro-CT images showing the planar cross-section of the submillimeter channel devices sintered and joined (**f**) at 1135 °C for 12 h and (**g**) 1150 °C for 6 h. Scale bar: 5 mm in photographs and 0.5 mm in SEM images, and 4 mm in micro-CT images.

The channel devices after joining at the different sintering conditions were cut to reveal the cross-section of the internal channels. The device joined at 1130 °C for 6 h (Fig. 6a) shows the gap in the bonding areas between the core part and bottom cover plate, indicating that either the temperature or the sintering duration was not adequate to uniformly join the parts. This is consistent with our earlier observation from the pull test of the joined block samples. The improved joining interface was seen for all other devices joined at temperatures equal to or higher than 1135 °C and are shown in Fig. 6b–d, where the bonding interface is not discernable. See Supplementary Information Figure S1 for the zoomed-in SEM image near the joining interface. Consistent with the mechanical test results of the joined blocks, these sintering conditions appear to produce better-quality joining interfaces.

Another important aspect to be considered when evaluating the joining quality is a degree of distortion in final channel shape. As shown in Fig. 6d, if we continue to increase the sintering temperature, the original channel shape is no longer retained. Shape distortion arises during the sintering/joining step mainly for two reasons. First, non-uniform shrinkage could take place as the surfaces of the parts in the assembly are subject to different boundary conditions. Green parts are highly porous after the binder burn-out step (only 50–70% dense^37^) and therefore significantly shrink upon sintering. Table 1 shows the dimensions of the channel devices sintered at various conditions. The overall shrinkage of the devices ranges from approximately 18–19% in the planar (width or length) direction to 20–22% in the z (build) direction. The observation that the BJP part shrunk more in the build direction compared to the other directions was also reported by other researchers^33^. This anisotropic shrinkage is more prominent for the channel features, i.e., more shrinkage was observed in the height than in the width with much larger variances. The average shrinkage observed in this study is also consistent with our earlier work^37^ on the SS/BN-based BJP parts, which reported an overall shrinkage of 15–25% as measured by a thermomechanical analyzer (TMA). Shrinkage at the mating surfaces or the joining interface of the device may be different from that of the other free surfaces, leading to the distortion of the overall assembly or individual channels.

**Table 1:** Dimensions of the submillimeter channel devices before and after sintering.

	Designed	Printed	Sintering condition			
			1130 °C, 6 h	1135 °C, 6 h	1135 °C, 12 h	1140 °C, 6 h
Device width	19.00	19.10	15.74 (17.6%)	15.65 (18.1%)	15.52 (18.7%)	15.56 (18.5%)
Device length	29.50	29.54	24.09 (18.4%)	23.88 (19.2%)	23.87 (19.2%)	23.71 (19.7%)
Device thickness	9.50	9.75	7.82 (19.8%)	7.56 (22.4%)	7.57 (22.3%)	7.66 (21.4%)
Channel height	2.00	2.11	1.63 ± 0.035 (22.7%)	1.53 ± 0.017 (28.9%)	1.75 ± 0.016 (17.1%)	1.50 ± 0.015 (27.5%)
Channel width	0.60	0.62	0.51 ± 0.093 (17.7%)	0.50 ± 0.045 (19.4%)	0.54 ± 0.018 (12.9%)	0.50 ± 0.043 (19.4%)

Measured from the cross-sectional SEM images (see Fig. 6). Unit = mm. Percentage in parenthesis indicates dimensional shrinkage.

Secondly, a high local concentration of sintering additives and/or high sintering temperature produces more liquid phase at the interfaces between the SS powder particles and the boron compounds (i.e., BN), rendering the part to change its shape^37^. Due to the significant size difference between the SS and BN powders, BN powder tends to surround the SS powder, forming liquid phase between two dissimilar powders. The sintering additive facilitates the sintering process and increases the part density and joining strength by enhanced consolidation, but the excessive sintering additives or high sintering temperature generate too much liquid phase, jeopardizing the intended shape of the final part. The extensive formation of the liquid phase in the part sintered at 1150 °C for 6 h, for instance, renders the rectangular channels in the device to lose their shape and become rounded or completely collapsed (see Fig. 6e). The effect of the sintering temperature on the shape distortion of the channels is also clearly shown in the micro-CT images—the device sintered at 1135 °C for 12 h display the relatively uniform shape along the 250-mm long channel (see Fig. 6f) while the deformed and collapsed channel walls are observed in the device sintered at 1150 °C for 6 h (see Fig. 6g), possibly blocking a flow passage. Note that the average fracture strength of the joined samples sintered at 1150 °C for 6 h is around 221 MP (see Supplementary Information Figure S2), which is similar to the fracture strength of the samples sintered at 1140 °C for 6 h (~ 224 MPa). We can conclude that the sintering temperature equal to or higher than 1140 °C does not improve the joining or part strength but can distort the channel shape. Based on these observations, we concluded that the sintering condition of 1135 °C for 12 h is the optimal sintering parameter for the given SS/BN powder mixture.

**Figure 7:**
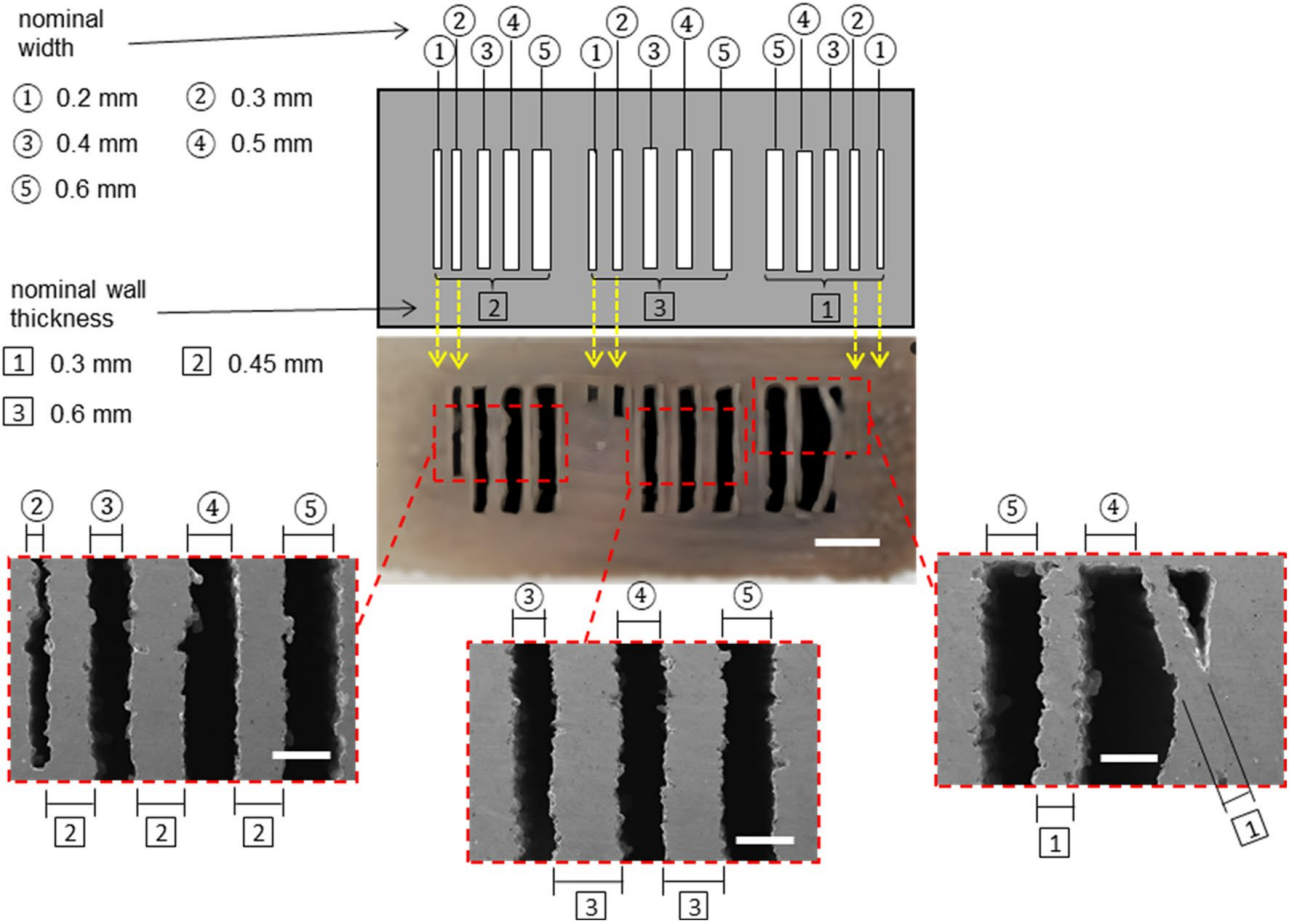
Schematic diagram and photograph of the varied channel widths and wall thicknesses (nominal dimensions indicated in mm) and SEM images with the measured dimensions in mm and shrinkage in %. This SS channel was sintered at 1135 °C for 12 h. Scale bar: photograph = 3 mm, SEM images = 0.5 mm.

Based on these optimized sintering parameters, we explored the minimum channel dimensions that can be achieved by the proposed BJP and joining approach. Generally, the printing resolution in the BJP process relies on the stock powder size, binder droplet size (determined by fluid properties of the binder phase and nozzle dimension), and print strategy (e.g., printing speed, part orientation)^33^. Using the BJP system similar to ours, researchers demonstrated the fabrication of microchannels or mesh structures as small as 250 μm^42^. Here, we designed and printed another channel device with a width varying from 0.2 to 0.6 mm and with a wall thickness from 0.3 to 0.6 mm. This device was sintered at 1135 °C for 12 h as it was determined to be the optimal sintering condition from the earlier experiments. If the channel wall is too thin, it will likely yield and become collapsed. Likewise, if the channel width is too narrow, it may not be resolved in printing or will be likely be clogged during sintering. As shown in Fig. 7 and Table S3 (see Supplementary Information), the minimum channel width and wall thickness needed for defect-free channel formation are 0.4 mm and 0.45 mm, respectively. The channels of 0.2 mm and 0.3 mm in width are completely or partially blocked regardless of the wall thickness as indicated in the yellow dotted lines in Fig. 7. The wall thickness of 0.3 mm appears unstable for this high-aspect-ratio channel and is likely to buckle during the sintering step. However, the same wall thickness may work satisfactory for the reduced channel height.

### Fluidic device measurements

**Figure 8:**
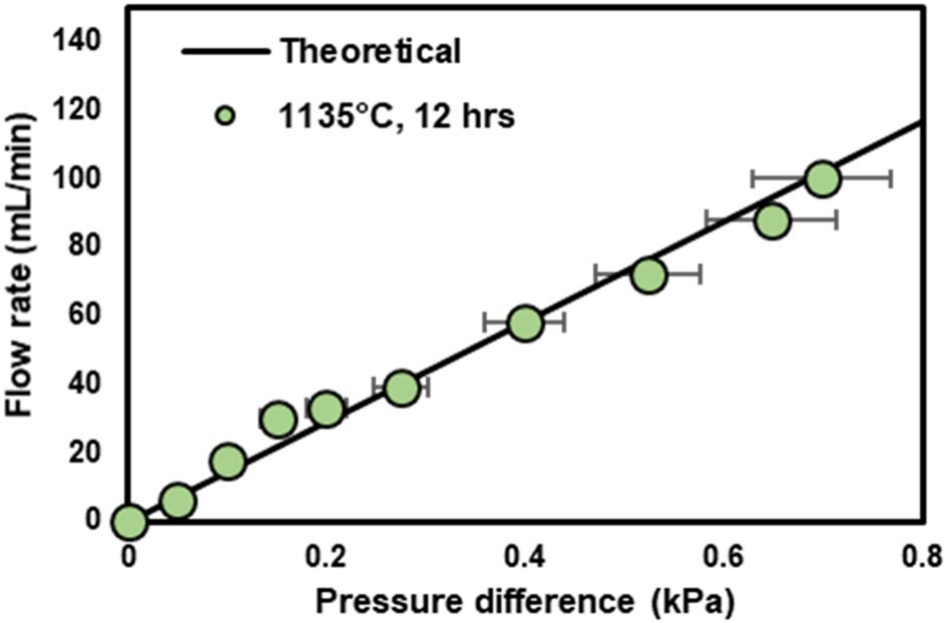
Volumetric flow rates of air measured as a function of pressure difference across the SS submillimeter channel device (sintered at 1135 °C for 12 h). The solid line indicates the Poiseuille flow model for the rectangular channel under the low-Re-number flow regime.

In our earlier works, we reported the relative density of 96–99% from the BJP-processed SS/BN cubes with the sintering conditions similar to this work^37,38^. The SS/BN microfluidic devices printed as a single part were sufficiently dense and did not show any leakage through the channel wall or fluidic connectors^29^. However, because the proposed channel device has been created using the joining process and it is difficult to ensure that all the joining interfaces are defect free (e.g., via micro-CT imaging), we need another experimental means to verify if there is any leakage or cross-talk between neighboring channels. Here we measure the pressure drop across both ends of the channel and compare it to a theoretical value. If there is any leakage (or blockage) in the channels, the measured pressure drop will be lower (or higher) than the theoretical value. For the theoretical investigation, we employ the well-known Poiseuille flow characteristics. Note that, under the pressure drop range investigated in this work (< 0.7 kPa), the air compressibility does not affect the flow behavior noticeably^43^. Furthermore, the secondary flow losses are negligible due to low Reynolds numbers (*Re* ~ O(10)). Under these circumstances, the serpentine channel can be replaced by a straight channel with the same cross-sectional dimensions and a length equal to the overall length of the serpentine. The pressure drop (*∆p*) versus volumetric flow rate (*Q*) for such a straight, and rectangular channel of height *H*, width *W*, and length *L*, filled with a fluid with a dynamic viscosity of *μ* can be expressed as^44^:

$$ \Delta P = \frac{{a\mu L}}{{WH^{3} }}Q $$

where $a = 12/[(1 - 0.63(H/W)]$. For pressure drop measurement, the device sintered at 1135 °C for 12 h was used with the following dimensions: *H* = 1.75 mm, *W* = 0.54 mm, and *L* = 0.4 m. The dynamic viscosity of air is μ = 1.8 × 10^−5^ Pa·s at room temperature. Theoretical and experimental results of the volumetric flow rates over the applied pressure difference of 0–700 Pa were obtained for air flows and are shown in Fig. 8. A good agreement between the experimental and theoretical results indicate that no significant defects such as channel blockage and leakage between the adjacent parallel channels exists in the fabricated device.

## Conclusion

In this paper, we introduced an innovative method for manufacturing a long, high-aspect-ratio, serpentine submillimeter channels made from stainless steel (SS) using binder jet printing (BJP) and liquid-phase sintering (LPS). Rather than constructing a microfluidic component as a single entity, our approach involves the printing of separate parts—two cover plates and one core part containing open channels—and the subsequent joining of these parts during the sintering step. Specifically, the core component forming the channels can be designed with one side open for easy powder removal. We leveraged liquid phase sintering—an addition of a small quantity of sintering additives (here boron nitride) helps to form liquid phase locally at the SS/BN powder interfaces and facilitate the simultaneous consolidation and joining of two or more SS green parts printed from BJP. The sintering temperature is a key process parameter that controls the amount of liquid phase for the optimal joining condition. From our systematic study, we found that the device sintered at 1135 °C for 12 h displayed the joining strength comparable to the bulk material while the shape distortion of the device assembly was kept to a minimum. Such submillimeter channel device was tested for pressure drop measurement of an air flow, demonstrating that the long serpentine channel was free of leaks, cross-talks, and clogs. Further work is geared towards performing more systematic tensile testing and microstructure analysis to reveal the process-property relationship for the proposed joining method.

### Supplementary Information

Supplementary Information.

## Data Availability

The datasets used and/or analysed during the current study available from the corresponding author on reasonable request.
