# Supplementary material for: Additively manufactured, long, serpentine submillimeter channels by combining binder jet printing and liquid-phase sintering
Source: Sci Rep. 2024 Jul 22;14:16825. doi: 10.1038/s41598-024-65058-5 (PMC11263481; doi:10.1038/s41598-024-65058-5)
Supplement: Supplementary file 1 — Supplementary Information. [file 41598_2024_65058_MOESM1_ESM.docx]

**Additively Manufactured, Long, Serpentine Submillimeter Channels by Combining Binder Jet Printing and Liquid-Phase Sintering**

Truong Do^1^, Hawke Suen^2^, Aryan Mehboudi^3^, Tyler Bauder^2^, Christopher Rudolf^4^, Patrick Kwon^2*^, Junghoon Yeom^4**^

**Supplementary Information**

Table S1. List of experimental parameters explored in this study

| Type of Device | Nominal Channel  Width  (mm) | Nominal Channel  Wall Thickness (mm) | Nominal Channel  Height  (mm) | Sintering Temperature (℃) | Sintering Time  (hour) |
| --- | --- | --- | --- | --- | --- |
| Sintering  Parameters | 0.6 | 0.6 | 2.0 | 1130 | 6 |
|  |  |  |  | 1135 | 6 |
|  |  |  |  | 1135 | 12 |
|  |  |  |  | 1140 | 6 |
| Channel  Dimensions | 0.2, 0.3, 0.4, 0.5, 0.6 | 0.3, 0.45, 0.6 | 2.0 | 1135 | 12 |


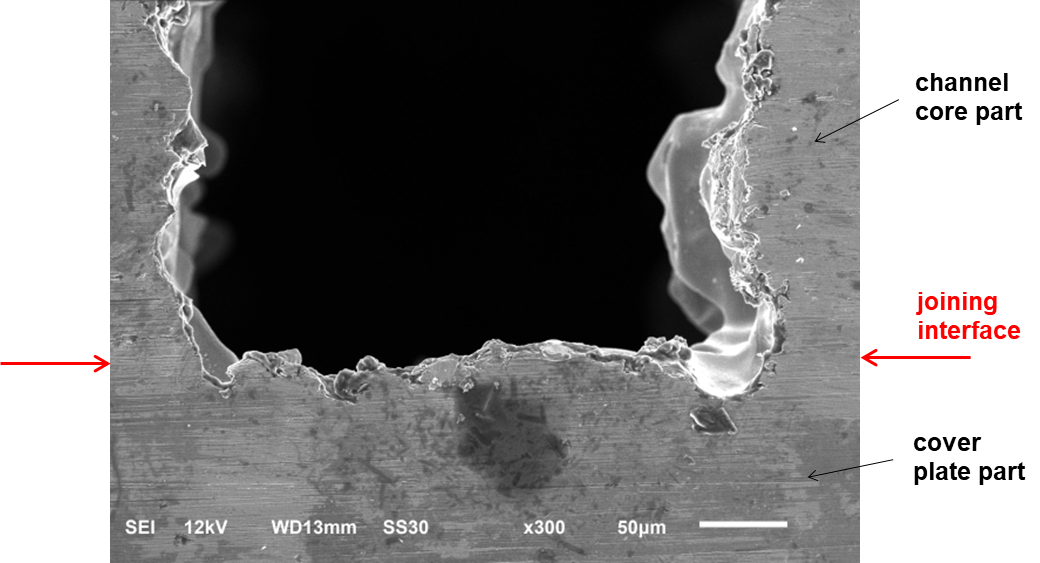


Figure S1. Zoomed-in SEM image of the SS submillimeter channel device sintered and joined at 1135°C for 12 hours, showing that the joining interface is not discernable.

The joining interface that supposedly exists between the channel core part and cover plate part is indicated by the red arrows. The interface is not discernable, i.e., no indication of separation or defect near the bonding regions. Note that visual inspection from a SEM image is limited by the resolution set by magnification. Considering that each pixel size in a SEM image with the magnification of x300 is around 0.35 µm, any defect smaller than 0.5 µm would be difficult to be detected.

Table S2. Size and cross-sectional areas of the joined samples for the strength characterization

| Sintering  Condition | Sample Number | Width  (mm) | Thickness (mm) | Cross-sectional Area (mm^2^) |
| --- | --- | --- | --- | --- |
| 1130°C, 6 hrs | 1 | 6.44 | 1.02 | 6.57 |
|  | 2 | 6.23 | 1.05 | 6.54 |
|  | 3 | 6.34 | 1.03 | 6.53 |
| 1135°C, 6 hrs | 1 | 6.52 | 1.07 | 6.98 |
|  | 2 | 6.61 | 1 | 6.61 |
|  | 3 | 6.6 | 1 | 6.6 |
| 1135°C, 12 hrs | 1 | 6.51 | 1.08 | 7.03 |
|  | 2 | 6.44 | 1.1 | 7.08 |
|  | 3 | 6.39 | 1.06 | 6.77 |
| 1140°C, 6 hrs | 1 | 6.35 | 1.05 | 6.67 |
|  | 2 | 6.43 | 1.05 | 6.75 |
|  | 3 | 6.34 | 1.04 | 6.59 |


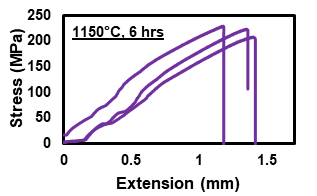


Figure S2. Stress vs. extension of the SS joined parts (three duplicates) sintered at 1150°C and 6 hours.

Table S3. Dimensions of the submillimeter channel devices to study the minimal channel width – sintered at 1135°C for 12 hours. Measured from the cross-sectional SEM images (see Figure 7). Percentage in parenthesis indicates dimensional shrinkage.

| Wall Thickness | | | Channel Width | |
| --- | --- | --- | --- | --- |
| Nominal Thickness (mm) | Wall Location  (between channel width) | Measured Wall Thickness  (mm) | Nominal Channel Width (mm) | Measured Channel  Width  (mm) |
| 0.3 | 0.2/0.3 | N/A | 0.2 | N/A  (fully closed) |
|  | 0.3/0.4 | N/A | 0.3 | N/A  (fully closed) |
|  | 0.4/0.5 | 0.22 ± 0.023 (buckled)  (26.7%) | 0.4 | N/A  (mostly closed) |
|  | 0.5/0.6 | 0.27 ± 0.04  (10%) | 0.5 | N/A  (open but deformed) |
|  |  |  | 0.6 | 0.47 ± 0.039  (21.7%) |
| 0.45 | 0.2/0.3 | N/A | 0.2 | N/A  (fully closed) |
|  | 0.3/0.4 | 0.31 ± 0.019  (31.1%) | 0.3 | 0.15 ± 0.035  (partly open) |
|  | 0.4/0.5 | 0.43 ± 0.025  (4.4%) | 0.4 | 0.33 ± 0.03  (24%) |
|  | 0.5/0.6 | 0.37 ± 0.016  (17.8%) | 0.5 | 0.44 ± 0.035  (12%) |
|  |  |  | 0.6 | 0.46 ± 0.032  (23.3%) |
| 0.6 | 0.2/0.3 | N/A | 0.2 | N/A  (mostly closed) |
|  | 0.3/0.4 | N/A | 0.3 | N/A  (mostly closed) |
|  | 0.4/0.5 | 0.54 ± 0.028  (10%) | 0.4 | 0.35 ± 0.026  (12.5%) |
|  | 0.5/0.6 | 0.49 ± 0.03  (18.3%) | 0.5 | 0.42 ± 0.033  (16%) |
|  |  |  | 0.6 | 0.46 ± 0.037  (23.3%) |
